# In vivo 3D brain and extremity MRI at 50 mT using a permanent magnet Halbach array

**DOI:** 10.1002/mrm.28396

**Published:** 2020-07-05

**Authors:** Thomas O’Reilly, Wouter M. Teeuwisse, Danny de Gans, Kirsten Koolstra, Andrew G. Webb

**Affiliations:** ^1^ C.J. Gorter Center for High Field MRI, Department of Radiology Leiden University Medical Center Leiden The Netherlands; ^2^ Technical University Delft Delft The Netherlands

**Keywords:** gradient design, Halbach array, low‐field MRI, permanent magnets, sustainable imaging

## Abstract

**Purpose:**

To design a low‐cost, portable permanent magnet‐based MRI system capable of obtaining in vivo MR images within a reasonable scan time.

**Methods:**

A discretized Halbach permanent magnet array with a clear bore diameter of 27 cm was designed for operation at 50 mT. Custom‐built gradient coils, RF coil, gradient amplifiers, and RF amplifier were integrated and tested on both phantoms and in vivo.

**Results:**

Phantom results showed that the gradient nonlinearity in the y‐direction and z‐direction was less than 5% over a 15‐cm FOV and did not need correcting. For the x‐direction, it was significantly greater, but could be partially corrected in postprocessing. Three‐dimensional in vivo scans of the brain of a healthy volunteer using a turbo spin‐echo sequence were acquired at a spatial resolution of 4 × 4 × 4 mm in a time of about 2 minutes. The T_1_‐weighted and T_2_‐weighted scans showed a good degree of tissue contrast. In addition, in vivo scans of the knee of a healthy volunteer were acquired at a spatial resolution of about 3 × 2 × 2 mm within 12 minutes to show the applicability of the system to extremity imaging.

**Conclusion:**

This work has shown that it is possible to construct a low‐field MRI unit with hardware components costing less than 10 000 Euros, which is able to acquire human images in vivo within a reasonable data‐acquisition time. The system has a high degree of portability with magnet weight of approximately 75 kg, gradient and RF amplifiers each 15 kg, gradient coils 10 kg, and spectrometer 5 kg.

## 1 INTRODUCTION

In the past few years there has been renewed interest in designing human MRI systems with field strengths significantly below those (1.5 T and 3 T) used for standard clinical examinations.^1^ Much of the rationale arises from the very high purchase, maintenance, and siting costs associated with such systems, all of which put them out of the financial reach of much of the world’s population. In addition to financial considerations, there are intrinsic advantages in terms of fewer patient contraindications and reduced specific absorption ratio compared with higher field systems.

Although there is a significant loss in SNR at low fields, the work by the Rosen group, in particular, has shown that diagnostically useful image quality can be achieved using fields as low as 6.5 mT using highly efficient sequences in combination with a very homogeneous magnetic field created by a large electromagnet.^2^, ^3^, ^4^ Representing a slight increase in hardware complexity, combinations of prepolarizing electromagnets and lower field either electro‐based or permanent magnet‐based readout systems have been used for imaging human extremities.^5^, ^6^ More sophisticated field‐cycling systems^7^, ^8^, ^9^, ^10^, ^11^ have also been used to obtain data over a wide range of field strengths, most recently in vivo data at 50 μT.

A fourth approach to low‐field MRI, which has the advantages of the relative simplicity of a single fixed field strength magnet, no requirement for a magnet power supply, and increased portability, is to use/design a permanent magnet. Although the original commercial C‐arm or H‐arm systems were large and heavy (>1000 kg) with field strengths of between 0.1 T and 0.35 T,^12^ more recently, a relatively inexpensive commercial system with significantly reduced size operating at 60 mT has shown very high quality images of the brain, with potential applications in the emergency room (www.hyperfine.io). However, even this system weights approximately 500 kg, which limits its transportability.

To reduce the weight of the magnet, a large number of distributed small magnets, rather than two very large disc magnets as in most permanent magnet designs, can be used. The most common geometry is a discretized version of a dipolar Halbach magnet,^13^ which is an optimal design in terms of producing the highest homogeneous magnet field within the structure and minimal field outside. This discrete geometry is in practice constructed from many small individual magnets. There are a number of papers in the literature that describe the design and construction of Halbach arrays for different applications, and analysis techniques for calculating both magnetic fields and interelement forces produced by such arrays.^14^, ^15^, ^16^ For NMR and MRI applications, the Halbach array was first demonstrated using the k = 1 dipolar mode, which produces a relatively homogeneous transverse magnetic field, in a series of papers from Blümler et al,^17^, ^18^, ^19^ who constructed magnets capable of measurements on small animals and plants. A number of other small‐scale systems have been constructed using modifications of this basic approach.^20^, ^21^, ^22^ Magnets with an in‐built linear gradient have also been proposed^23^ and designed,^24^, ^25^ in which rotation of the magnet around the sample enables 2D spatial information to be encoded and reconstructed. More recently, such systems have eliminated the need for rotating the system by adding two coil‐based phase‐encoding gradients to achieve 3D imaging.^26^, ^27^ Arrays of permanent magnets can also be arranged in a so‐called inward‐outward pair, in which the magnets in two rings are arranged with their magnetization pointing radially inward and outward in respective rings, generating a B_0_ field that is oriented along the bore, more closely resembling conventional MRI systems.^28^, ^29^

Recently, our group described the design, construction, and characterization of a larger 27‐cm clear‐bore Halbach array magnet operating at 50 mT.^30^ A genetic algorithm was used to optimize the geometry of the 23‐ring magnet, and the inclusion of additional shimming rings to compensate for material and construction imperfections resulted in a homogeneity of approximately 2400 ppm over a 20‐cm diameter of spherical volume. Using simple gradient coils, 3D spin‐echo images at 3.5 × 3.5 × 3.5 mm spatial resolution were acquired in an imaging time of about 35 minutes from a phantom. However, this time is much too long for a useful clinical scan; in addition, the gradient coils had too high a resistance to be able to implement efficient imaging sequences with a high duty cycle. Because our major aim for a portable MRI system is to image pediatric hydrocephalus in resource‐limited locations, we ultimately need the system to acquire images distinguishing between CSF and white matter and gray matter. The role of imaging in pediatric hydrocephalus has recently been reviewed.^31^ As described in this review and earlier papers,^32^ 3D T_1_‐weighted and T_2_‐weighted images form the essential anatomical sequences, with other sequences supplementing these based on the particular feature of the disease being studied. Although much higher spatial resolution can be achieved at high fields, the loss in SNR at low fields can be retrieved partially by the use of long echo trains in turbo spin‐echo sequences due to the long T_2_ value of CSF and the very low specific absorption rate intrinsic to low‐field MRI.

In this paper we design a long echo‐train turbo spin‐echo sequence to image the CSF in healthy adult brain. The Cartesian k‐space coverage is restricted to an elliptical coverage to reduce the data acquisition time to less than 3 minutes for a 4 × 4 × 4 mm isotropic spatial resolution. We also show applications for extremity imaging, in which higher resolution (~3 × 2 × 2 mm) in vivo images of the human knee are captured in just over 10 minutes. Issues of temperature drift and image reconstruction in the presence of gradient nonlinearities are also discussed.

## 2 METHODS

### 2.1 Magnet design

Details of the magnet design have been published previously^26^ and are minimally described here. The Halbach magnet consists of 23 rings, with two layers of N48 neodymium boron iron magnets (12 × 12 × 12 mm^3^) per ring. The array has a clear bore of 27 cm and a length of 50 cm between the two outer rings. The ring diameters were designed using an optimization scheme to give the highest B_0_ homogeneity; the source code is available at https://github.com/LUMC‐LowFieldMRI/HalbachOptimisation. After measuring the field using a 3D robotic device, additional shimming was implemented by defining a 32.5‐cm‐diameter, 28‐cm‐long cylindrical grid of 15 rings of 60 potential magnet positions per ring. The filling of this grid was subsequently optimized using a genetic algorithm that has previously been shown to be suitable for creating tailored magnetic fields using permanent magnets,^24^ which had three options for each magnet position: no magnet, a magnet following the k = 1 Halbach orientation, and the magnet flipped 180º from that orientation. The genetic algorithm minimizes the field variation over a 20‐cm‐diameter spherical volume with 3 × 3 × 3 mm N45 neodymium boron iron magnets being used to fill the grid. The optimal solution occupied approximately 600 of the 900 grid positions, and once constructed reduced the field inhomogeneity from 13 000 ppm to 2400 ppm, with most of the B_0_ deviations located toward the edges of this diameter of spherical volume along the z‐axis. The total weight of the magnet, including all components, is about 75 kg.

**FIGURE 1:**
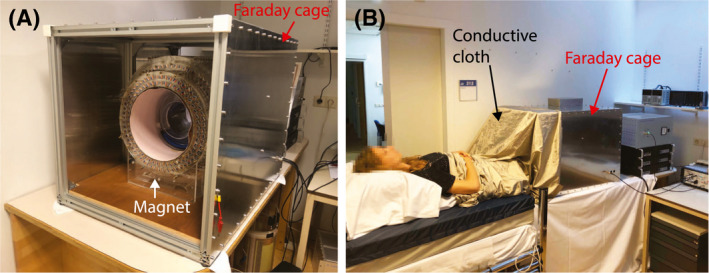
A, Magnet setup. The shims and gradients are integrated into the bore of the magnet. The entire setup is placed inside an aluminum Faraday cage to reduce environmental noise in the setup. B, A conductive cloth is placed over the subject to reduce the noise coupled into the system through the body

To minimize the environmental noise, the entire setup was placed inside a 62.5 × 62.5 × 85 cm Faraday cage constructed from 2‐mm‐thick aluminum sheets and 32 × 32 mm^2^ aluminum extrusion profiles. Because the body can act as a very effective antenna, such as coupling to power lines in the surrounding walls,^21^ the torso extending out of the Faraday shield was placed under a conductive fabric (Holland Shielding Systems, Dordrecht, the Netherlands). No direct contact between the skin and the conductive cloth was required for the shield to be effective. The sheet was connected to the same ground as the Faraday cage, as shown in Figure 1.

### 2.2 Radiofrequency coil

For the phantom and knee imaging experiments, a 15‐cm‐long, 15‐cm‐diameter solenoid with 57 windings was used as a transmit/receive coil. The solenoid was segmented using three nonmagnetic capacitors (47 pF; American Technical Ceramics E‐series, Huntington Station, NY) spaced evenly along the length of the solenoid to avoid self‐resonance effects. Impedance matching used an L‐network with 33 pF and 136 (2 × 68) pF capacitors in the parallel and series arms, respectively. There was a 4‐kHz shift downward in the resonant frequency when the knee was inserted. The Q value decreased from 54 to 47 when the coil was loaded with the knee, indicating that coil loss is dominant, as expected.^33^ A thin copper sheet (50 µm) was placed inside the gradient coils to reduce the interaction between the self‐resonant modes of the gradient coils and the RF coil, as well as to reduce noise coupled into the system from the gradient amplifier. Six 800‐pF chip capacitors were placed along the seam of the sheet to reduce eddy currents induced by switching of the gradients. The resonance frequency of the RF coil increased 95 kHz when the coil was inserted into the magnet.

For in vivo brain imaging, an elliptical solenoid was constructed with a width of 18.5 cm, height of 24.5 cm, and length of 20 cm, with 40 windings of 1‐mm diameter copper wire. It is segmented in three places with 82‐pf capacitors, with a 1‐pF to 30‐pF variable capacitor placed in parallel with one of the segmentation capacitors. Impedance matching used an L‐shaped matching network with 101 pF (68 pF + 33 pF) and 150 pF (82 pF + 68 pF) capacitors in the parallel and series arms, respectively. The resonance frequency increased 233 kHz when the coil was inserted into the magnet. The unloaded Q was 88, and loading the coil with a human head resulted in a 4‐kHz decrease in the resonance frequency of the coil and a decreased Q of 78.

When either coil was used without the conductive sheet in place, the noise level increased by approximately a factor of 10 due to the “body antenna” effect. When the sheet was carefully wrapped around the entire body, and physical contact with the Faraday shield was present at several points, the noise level was essentially unchanged from a coil loaded with a tissue‐mimicking phantom within the solid Faraday enclosure.

### 2.3 Three‐axis gradient coils

In a previous publication,^30^ simple quadrupolar Y and Z gradients were constructed. These had high inductance (203 µH) and very high resistance (10.0 Ω). Here, new gradient coils with improved performance were designed, constructed, and integrated. Gradient coils were designed using the target field approach^34^ adapted to produce their encoding fields commensurate with the transverse B_0_ direction of the Halbach array. This results in y‐gradient and z‐gradient coils that are similar in design to a quadrupolar design, but an x‐gradient coil with significantly improved performance compared with a simple saddle design (full mathematical details are provided in de Vos et al^35^).

**FIGURE 2:**
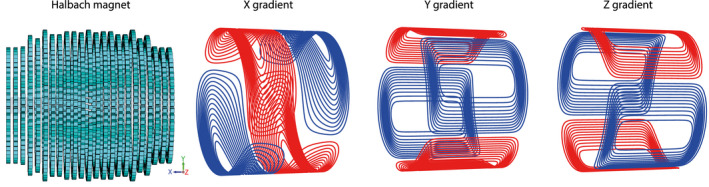
Three imaging gradients are designed using the target field method, adapted for the Halbach configuration with the B_0_ field oriented across the magnet bore (left schematic); the line color indicates the direction of current flow. The gradients were constructed using copper wires placed into a 3‐mm‐thick 3D printed structure

Gradients coils were constructed using 1.5‐mm‐diameter copper wire pressed into 3D printed formers. The efficiencies of the x‐gradient, y‐gradient, and z‐gradient coils were 0.59, 0.95 and 1.02 mT/m/A, respectively. The inductance of the x‐gradient was 180 µH with a resistance of 0.37 Ω, and the inductances of the y‐gradient and z‐gradient were both 225 µH with resistances of 0.4 Ω. Schematics of the wire layouts for the three gradient coils are shown in Figure 2, 3. Three‐dimensional models for the x‐gradient, y‐gradient, and z‐gradient coils are given in Supporting Information Figures [Link], [Link], [Link], respectively.

**FIGURE 3:**
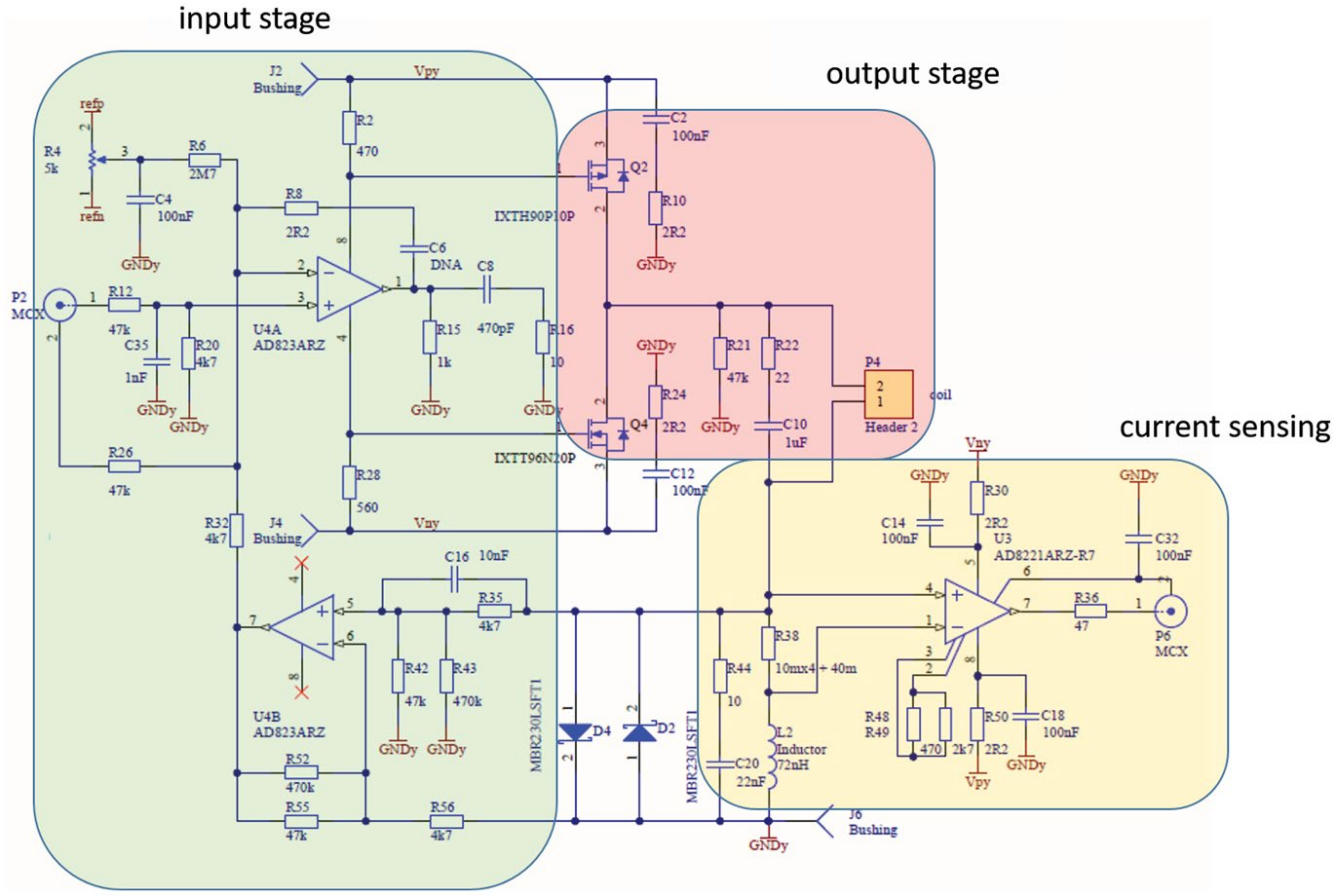
The three modules used in each of the three gradient amplifiers channels

### 2.4 Gradient amplifiers

Gradient power amplifiers (GPAs) on most clinical 1.5T and 3T superconducting whole‐body scanners have maximum gradient strengths in the range of 40‐80 mT/m, with slew rates of 150‐200 T/m/s. The GPAs for these systems are capable of simultaneously generating a driving current of 1 kA and voltage of 2 kV over a bandwidth of tens of kHz by cascading the outputs of several pulse‐width modulated stages. Typically the current signal is controlled by active feedback using high‐precision current sensors built into the circuit. Gain accuracy and linearity are typically below 0.05%, and total harmonic distortion below 0.25%.^36^ The GPAs for preclinical imaging are typically four‐quadrant linear designs with maximum output currents on the order of 40‐80 A, with maximum voltages of 100‐200 V. These GPAs can also be used for MR microscopy. In one example, Seeber et al described a pulsed current gradient power supply for very high spatial resolution microimaging.^37^

However, for lower field systems aimed at creating low‐cost, low‐maintenance MRI systems, such high gradient performance is not required. In this type of system, spin echo–based sequences with TEs on the order of tens of milliseconds indicate that very rapid switching and rise times (high slew rates) are not required. Spatial resolutions on the order of several millimeters indicate that frequency‐encoding gradient strengths on the order of tens of millitesla per meter are sufficient, and much more inexpensive linear GPA designs can be used. Wright et al developed a desktop MRI system capable of imaging objects approximately 2 cm in diameter. The GPAs used LM12 high‐power op‐amps capable of driving ±10 A into gradient coils with an efficiency of 0.45 G/cm/A.^38^ One recent example is the system constructed to run educational MRI devices: The OpenMRI project uses two OPA 549 power op‐amps (Texas Instruments, Dallas, TX) in a bridged configuration, with a maximum output current of 8 A.^39^

Because we do not need very high currents to achieve the desired spatial resolution (~3 mm,) high currents are not necessary, and the maximum voltage (15 V) is chosen such that simple air‐fan and heatsink cooling can be used. Each amplifier was constructed using a push‐pull configuration. The design for each channel is shown in Figure 3. The basic design is a pair of bridged operational amplifiers (U4A and U4B), which provide voltage gain to the input of a rail‐to‐rail power MOSFET amplifiers. The MOSFETs are used in a common source configuration, which by approximation makes the low‐frequency loop gain and the output current independent of the load impedance. The gate voltage does not have to change to maintain the desired current at a different or changing output voltage. A common drain configuration would not have this advantage because its gate voltage then directly depends on the output voltage. Stability, however, does depend on the load impedance, because the load inductance and the C_gd_ of the MOSFETs form two dominant poles. Simulation shows stable operation from 10 μH up to 500 μH. For simplicity, the circuit is shown as three separate, but connected, modules, each of which is described in detail subsequently.

The input stage consists of an operational amplifier, which provides the necessary voltage gain sufficient to drive the MOSFETS. The output stage consists of a MOSFET rail‐to‐rail common source stage. It consists of two power MOSFETs arranged in a push‐pull configuration. The output MOSFETs are capable of delivering more than 90A and have a RDS ON of 25 mΩ. The fast intrinsic diodes of these O‐(2‐[^18^F]fluoroethyl)‐L‐tyrosines are capable of conducting current from the gradient coil back into the power supply. Their TO247 package has a large surface area that results in a low thermal resistance. L2 creates a phantom zero in the feedback loop that moves the poles of the amplifier to a better position for stability and sets the closed‐loop bandwidth of the amplifier to about 7 kHz. R22+C10 will damp the self‐resonance of the gradient coil. This helps stabilizing the amplifier further for a wide range of inductances. The instrumentation amplifier (AD8221ARZ) is a classic three op‐amp design, with 80‐dB common mode rejection ratio up to 10 kHz (unity gain) and a 2‐V/μs slew rate, with 8 nV/root Hz noise at 1 kHz.

**FIGURE 4:**
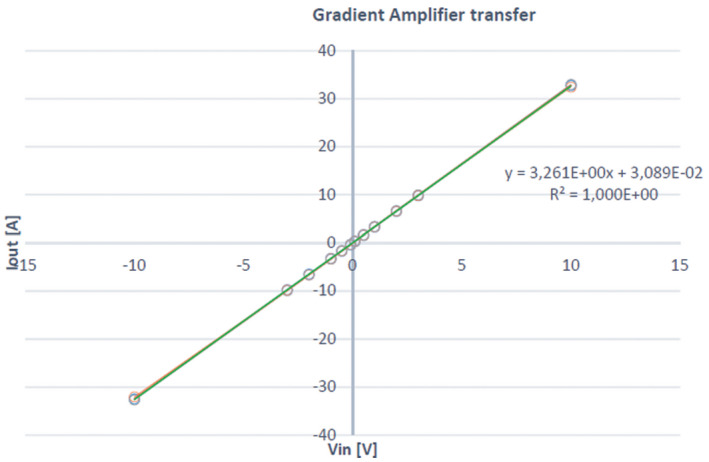
Plot of the linearity of the output current of the gradient amplifier as a function of the voltage supplied by the digital‐to‐analogue boards of the MR spectrometer

The current slew rate is a function of the maximum voltage (15 V), maximum current used (10 A, although the full range of the amplifier is up to 30 A), and coil inductance and resistance, and was measured to be approximately 30 T/m/s. Each gradient amplifier produces an output gain of 3.3 A per 1‐V input from the ±10 V 16‐bit digital‐to‐analogue gradient drivers of the MR console. Figure 4 shows the gain as a function of input voltage, indicating a linearity of the output current as a function of input voltage of less than 1%. First‐order resistor–capacitor filters (f_c_ = 7 kHz) were placed on the gradient lines to reduce RF noise introduced by the gradient amplifiers and gradient lines entering the Faraday cage.

A Magritek Kea2 spectrometer (Aachen, Germany) was used to generate gradient wave forms and RF pulses as well as digitizing the generated echoes. The RF pulses generated by the spectrometer were amplified by a custom‐built 1‐kW RF amplifier (schematics are provided in Supporting Information Figure S1).

### 2.5 Phantom studies

Images were acquired from a phantom consisting of forty‐five 35‐mm‐long, 8‐mm‐diameter tubes filled with water arranged on 3D‐printed 7 × 7 rectangular grid (four corner holes empty) with the center of the tubes spaced 17 mm apart. Data were acquired using a 2D spin‐echo projection sequence with the following scan parameters: FOV = 256 × 256 mm, 128 × 128 data points, TR/TE = 6000 ms/100 ms, and pulse duration = 100 µs. Images were acquired with the main axis of the phantom in the +y (vertical) and +x (horizontal) axes, both perpendicular to the B_0_ field.

### 2.6 In vivo imaging protocols

All images were acquired from healthy volunteers giving informed consent according to the policies of the local ethics committee.

For brain imaging, all data were acquired with a bandwidth of 20 kHz, a 90º pulse length of 100 μs with a power of 47 dBm, and a 180º pulse length of 100 μs with a power of 50 dBm. Phase cycling of successive 180º pulses in the turbo spin‐echo train followed the regular Carr‐Purcell‐Meiboom‐Gill modulation. k‐Space acquisition was restricted to an elliptical coverage in the two phase‐encoding directions, to reduce scan time. All data were filtered using a sine‐bell squared filter. B_0_ homogeneity over the brain, measured as the FWHM of the Fourier transform of the FID signal, was approximately 600 Hz.

Because the aim of the work is to map areas of CSF, seen mostly as bright areas on a darker background, we used a T_2_‐weighted sequence with long effective TE. Based on a series of low‐resolution inversion‐recovery images with TI varying between 100 ms and 2800 ms, the T_1_ of CSF was estimated to be about 1750 ms, with the T_2_ value assumed to be only slightly lower, as it is well‐known that at very low field T_1_ ≈ T_2_ for mobile liquids. Both the T_1_ and T_2_ relaxation times of brain tissue are much shorter, being reported on the order of 500 ms and 250 ms, respectively.^40^ Three types of images were run: T_2_‐weighted for bright CSF, T_1_‐weighted for a rapid anatomical image (CSF dark), and an inversion‐recovery sequence that produces good contrast but measurable signal intensities from both CSF and brain tissue. The spatial resolution was chosen to fulfill the relatively coarse requirements for pediatric imaging (hydrocephalus) within a very short imaging time of 2 minutes per 3D data set.

The sequence parameters for the sequences are as follows. For T_1_‐weighted imaging, FOV = 192 × 192 × 256 mm, acquisition matrix = 48 × 48 × 64, TR/TE = 500 ms/10 ms, echo train length (ETL) = 6, and acquisition time = 2 minutes 23 seconds (k‐space data were acquired on a center‐out trajectory); for T_2_ weighted imagine: FOV = 160 × 192 × 256 mm, acquisition matrix = 40 × 48 × 64, TR/TE = 3000 ms/20 ms, ETL = 40, effective TE = 410 ms, and acquisition time = 1 minutes 48 seconds (k‐space data were acquired on a low‐high linear trajectory); and inversion recovery: FOV = 160 × 192 × 256 mm, acquisition matrix = 40 × 48 × 64, TI/TR/TE = 100 ms/3000 ms/20 ms, ETL = 40, acquisition bandwidth = 20 kHz, and acquisition time = 1 minutes 48 seconds (k‐space data were acquired using a center‐out trajectory).

In addition to these relatively low‐resolution images, we also acquired a higher resolution image to demonstrate that the SNR is sufficient to acquire images at this resolution (FOV = 256 × 256 × 256 mm, acquisition matrix = 64 × 128 × 128, TR/TE = 500 ms/20 ms, ETL = 4, acquisition bandwidth = 20 kHz, and acquisition time = 13 minutes 2 seconds). k‐Space data were acquired on a center‐out trajectory.

For an illustrative case of knee imaging, in which higher spatial resolution is desirable and imaging times can be increased somewhat, we imaged the knee of a healthy volunteer. Relaxation data for muscle, fat, and cartilage were estimated from previously published formulas and measurements.^41^ The estimated T_1_ values were 200, 140 and 70 ms, and T_2_ values were 47, 84 and 20 ms, respectively. To obtain signal from all tissues (with no attempt to optimize contrast between tissues), data were acquired using a 3D T_1_‐weighted turbo spin‐echo sequence with the following parameters: FOV = 256 × 256 × 256 mm, acquisition matrix = 128 × 128 × 128, TR/TE = 115 ms/15 ms, ETL = 3, acquisition bandwidth = 20 kHz, and RF pulse duration = 100 µs. k‐Space data were acquired on a center‐out trajectory and filtered using a sine‐bell squared filter. B_0_ homogeneity over the knee, measured as the FWHM of the Fourier transform of the FID signal, was about 400 Hz.

### 2.7 Gradient nonlinearity correction

Fourier‐based image encoding assumes a linear relationship between space and gradient magnitude. Because of the relatively low length‐to‐diameter ratio (~1:1) possible for our gradient coils, there are significant image distortions arising from gradient nonlinearity, particularly along the x‐axis (along the bore of the magnet). A basic distortion correction is implemented by replacing the standard inverse fast Fourier transform by

$$\rho \left(x,y,z\right)=\sum_{x}\sum_{y}\sum_{z}s\left(k_{x},k_{y},k_{z}\right)e^{i2\pi k_{x}G_{x}\left(x,y,z\right)}e^{i2\pi k_{y}G_{y}\left(x,y,z\right)}e^{i2\pi (G_{z}\left(x,y,z\right)+\Delta B_{0}\left(x,y,z\right))t},$$

where ρ is the reconstructed proton density; $G_{x}$,$G_{y}$, and $G_{z}$ are simulated gradient field maps (from Biot‐Savart calculations using the wire geometries); and *t* is the readout time. This approach to gradient nonlinearity correction is analogous to that used in conjugate phase reconstruction,^42^ which is known to break down for large deviations in the encoding fields. In such cases, model‐based reconstructions have been shown to be more effective,^43^ although they are significantly more computationally expensive. The algorithm was implemented using *Python* 3.7 and *Numpy* 1.17. The optimized einsum function of *Numpy* was used to reduce memory consumption and reconstruction time. Reconstruction time for a 128 × 128 × 128 voxel data set was 43 seconds on an Intel Core i7‐6700 quad‐core CPU with 32 GB RAM. The algorithm was also adapted to run on a GPU using the CuPy Python library. Reconstruction time for a 128 × 128 × 128 voxel data set when executed on a Nvidia GTX 1660 super with 6 GB on‐board RAM was less than 1 second.

## 3 RESULTS

**FIGURE 5:**
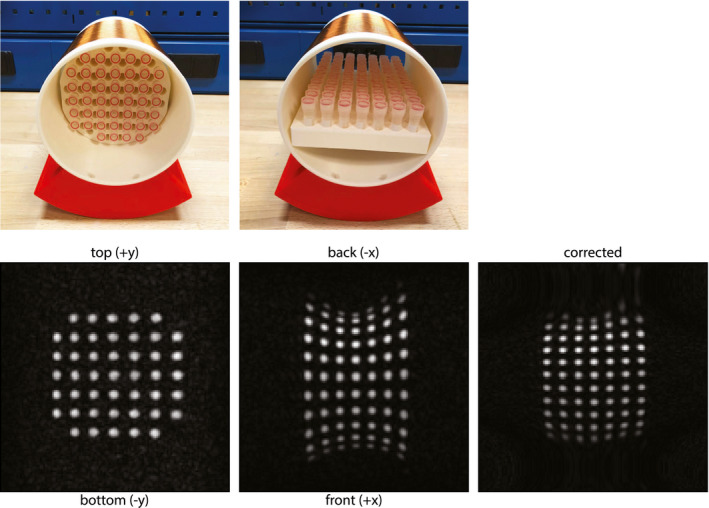
Images acquired of a phantom of tubes (8 mm diameter, 30 mm long), each placed 17 mm apart on a rectangular grid. Minimal distortions are seen in the central transverse plane; more significant distortions are present in the central coronal plane and are caused primarily by nonlinearities in the x‐gradient field

Figure 5 shows 2D projection phantom images, which illustrates the degree of geometric distortion particularly along the x‐axis due to the nonlinearity, and eventual compression point of the x‐gradient coil. Also shown is the result of the correction algorithm, which improves the reconstruction along this axis quite considerably.

**FIGURE 6:**
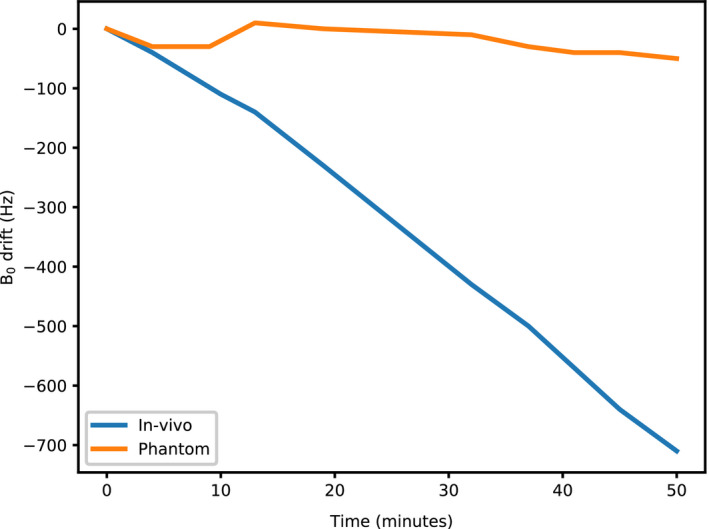
Measurement of the B_0_ drift measured spectroscopically during the phantom and in vivo experiments. Data were acquired over the entire sample within the active volume of the RF coil

Figure 6 shows a plot of the main field drift during experiments, measured from the resonance frequency of the proton spectrum acquired from the whole sample. For the phantom it is clear that the gradients produce negligible heating (and therefore field drift) in the experiment, so correction does not need to be applied. For the in vivo data, the heating introduced by the body is much greater. The rate of drift is approximately 800 Hz per hour, corresponding to 160 Hz over our in vivo imaging time of 12 minutes.

**FIGURE 7:**
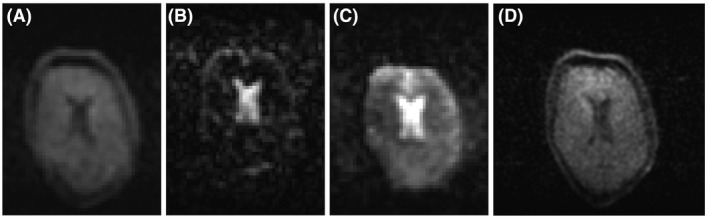
Images acquired with different weighting at a spatial resolution of 4 × 4 × 4 mm: T_1_‐weighted (2.5‐minute data‐acquisition time) (A); T_2_‐weighted (2 minutes) (B); inversion‐recovery turbo spin‐echo sequence (2 minutes) (C); and higher resolution image (2 × 2 × 4 mm) from a different volunteer (13 minutes) (D)

Figure 7 shows in vivo images of the brain of a healthy volunteers, with a slice through the center of the ventricles shown, as this is the area of most interest with respect to applications in hydrocephalus.

**FIGURE 8:**
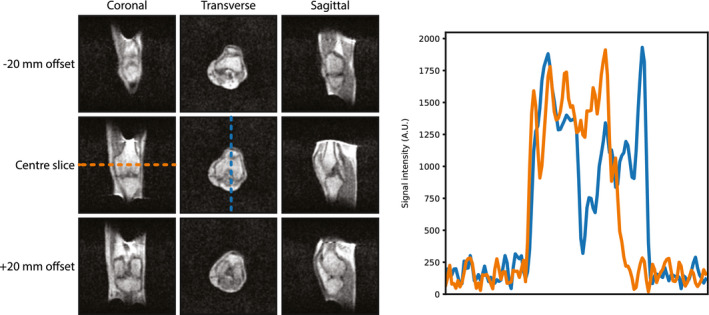
Left: Coronal, transverse, and sagittal (reformatted from a single 3D acquisition) knee images using a 3D turbo spin‐echo sequence with the following parameters: FOV = 256 × 256 × 256 mm, acquisition matrix = 128 × 128 × 128, TE/TR = 10 ms/130 ms, and acquisition time = 11 minutes 50 seconds. No gradient distortion correction has been applied to the images. Right: Projections taken through two of the central axes of the images

Figure 8 shows in vivo images of the knee acquired from a healthy volunteer. A single 3D data set was acquired, and the images shown are three central and 20‐mm offset planes through the data set.

**FIGURE 9:**
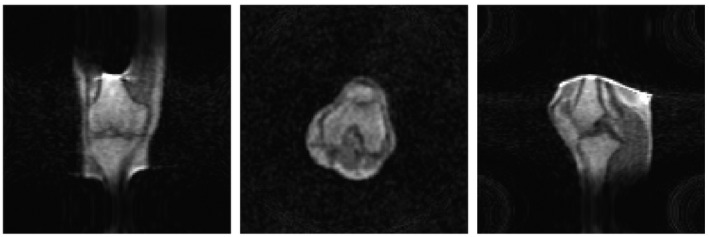
Central slices after applying the gradient correction algorithm. The remaining distortions in the image are located in areas of very strong gradient nonlinearity and reversal points for which the algorithm is unable to compensate

Figure 9 shows the results of the gradient nonlinearity correction, which shows improvement in the center of the FOV, but as expected is unable to deal with the severe nonlinearities close to the gradient null and reversal points at either ends of the gradient coil.

## 4 DISCUSSION

We have designed and constructed a low‐cost, portable, low‐field MRI system operating at 50 mT for in vivo imaging. We would characterize the novelties of the problem of compactness, low cost, and sustainability as follows:

1. Being able to design a permanent magnet array with readily available commercial small magnets that are inexpensive (ie, do not require very fine tolerances);
2. Being able to form the housing for such an array with readily available and inexpensive plastics, which can be readily machined with acceptable tolerances;
3. Potentially being able to repair the magnet very simply by replacing individual magnets or rings of magnets should damage occur;
4. Having each of the electronics components be run from standard power outlets, and ultimately be battery/solar/diesel engine powered; and
5. Designing gradient and RF amplifiers that operate for the specific tasks required (gradient slew times, operating frequency) rather than being multipurpose, much more expensive and heavy units.

In terms of power consumption and requirements, the RF amplifier runs off standard 110/240 V main supply and has to supply about 500 W at a duty cycle of less than 1%. The gradient amplifier also runs off a standard 110/240 V main supply and has to provide 300 VA at a duty cycle of about20%. In terms of heating, the RF amplifier and gradient amplifier both contain heat sinks and have simple air fan cooling. A thermal camera indicated that the maximum component temperature reached in the gradient amplifier was approximately 60°C.

In vivo images of the brain and the knee have been acquired. For the brain scans, our ultimate aim is to image pediatric hydrocephalus in resource‐limited settings. As discussed by Obungoloch et al,^44^ the required spatial resolution is not high by conventional standards, and so we determined how rapidly it is possible to acquire images with sufficient spatial resolution. These were acquired within a couple of minutes, with sufficient contrast between brain tissue (white matter/gray matter) and CSF, to be able to determine areas of fluid accumulation.

This type of system can also be used for applications in which the imaging time can be increased, and could be used as a point‐of‐care device. As an example, we showed that we can acquire in vivo images of the human knee with a nominal 2 × 2 × 2 mm resolution with a SNR of about 20:1 within 12 minutes.

To estimate the true resolution, there are several components in addition to digital resolution that must be considered. These include frequency drifts due to temperature changes (in vivo and gradient induced), the degree of B_0_ inhomogeneity over the imaging region of interest with respect to the strength of the frequency encoding gradient, and T_2_ decay during the turbo spin‐echo train. Taking each in turn, temperature‐induced B_0_ drift causes by heat radiated from the body (the thermal sensitivity, due to the temperature sensitivity of the magnet remanence, is approximately 2.5 kHz per degree Celsius) were measured for the knee scans to be approximately 160 Hz over the 12‐minute imaging session, which corresponds to a FWHM of the point spread function in the frequency‐encoding dimensions of around 1.9 mm for the 240‐mm FOV and 20‐kHz bandwidth used. However, these are very easily corrected by applying a corresponding phase shift to the k‐space data. The NMR‐measured inhomogeneity over the knee corresponded to a FWHM of about 400 Hz: Imaging with a pixel bandwidth of 300 Hz/pixel gives a point spread function of 1.3 pixels, or 2.6 mm in the frequency‐encoding dimension. The echo train length of three echoes with an effective TE of 15 ms and estimated T_2_ of muscle of about 200 ms means that the point spread function due to T_2_ decay during the echo train is subpixel^45^ in the corresponding phase‐encoding direction. Combining the effects of sampling and linewidth gives an estimated resolution of 3.3 mm in the frequency‐encoding direction, and about 2 mm in each of the phase‐encoding direction. Of course the former resolution could be reduced using a higher readout bandwidth per pixel, at the cost of reduced SNR, using the gradients to perform first‐order B_0_ shimming and including knowledge of the B_0_ field distribution in the image reconstruction. Similar calculations can be applied to the brain data, but because these are acquired at a coarser digital resolution of 4 × 4 × 4 mm, there is a correspondingly smaller effect. Several methods can also potentially be used to reduce the total imaging time, such as simple half Fourier, compressed sensing and parallel imaging, using multiple receivers.

Noise measurements performed with each of the components of the system present and absent indicate that there are some improvements still to be made in terms of SNR. With a 50‐Ω terminated input to the Magritek spectrometer, the noise level is 0.18 μV for a 100‐kHz bandwidth (the rms noise level is compared with an internal 1‐μV reference signal produced by the spectrometer), corresponding to 0.7 nV/square root Hertz, which is within experimental error of the theoretical limit of 0.9 nV/square root Hertz. This level remains unchanged when the 50‐Ω load is located at the end of a long coaxial cable, which is passed through the Bayonet Neill–Concelman interface to the inside of the Faraday cage. When the impedance‐matched RF coil is connected, the noise level is unchanged. Connecting the RF amplifier introduces no additional noise, but when the gradient amplifier is turned on, the noise level increases to 0.33 μV. This indicates that some degree of coupling exists between the gradient coils and RF coil. The first‐order gradient filter reduces this by approximately 10%, but there is still room for improvement in reducing the noise level. We also note that there are a series of harmonic spikes that occur every 200 kHz, arising from the gradient amplifier; however, given the small imaging bandwidth used, this is not a problem with the current setup.

We also anticipate that the Halbach magnet geometry can be expanded for future applications, moving away from the very simple cylindrical symmetrical structure illustrated here, such as hemispherical^46^ or linear geometries^47^ that might be appropriate for breast and adult head, and spine imaging, respectively.

## Supporting information


**FIGURE S1** Schematic of a custom‐built 1‐kW RF amplifier with the three main stages of the amplifier labeled

**FIGURE S2** A 3D model of the x‐gradient coil wire pattern designed using the target field method described in Krishnan et al^31^

**FIGURE S3** A 3D model of the y‐gradient coil wire pattern designed using the target field method described in Krishnan et al^31^

**FIGURE S4** A 3D model of the z‐gradient coil wire pattern designed using the target field method described in Krishnan et al^31^

## Data Availability

The ring diameters were designed using an optimization scheme to give the highest B_0_ homogeneity; the source code is available at https://github.com/LUMC‐LowFieldMRI/HalbachOptimisation.
