# Supplementary material for: In vivo 3D brain and extremity MRI at 50 mT using a permanent magnet Halbach array
Source: Magn Reson Med. 2020 Jul 5;85(1):495–505. doi: 10.1002/mrm.28396 (PMC7689769; doi:10.1002/mrm.28396)
Supplement: Supplementary file 1 — FIGURE S1 Schematic of a custom‐built 1‐kW RF amplifier with the three main stages of the amplifier labeled [file MRM-85-495-s001.pdf]

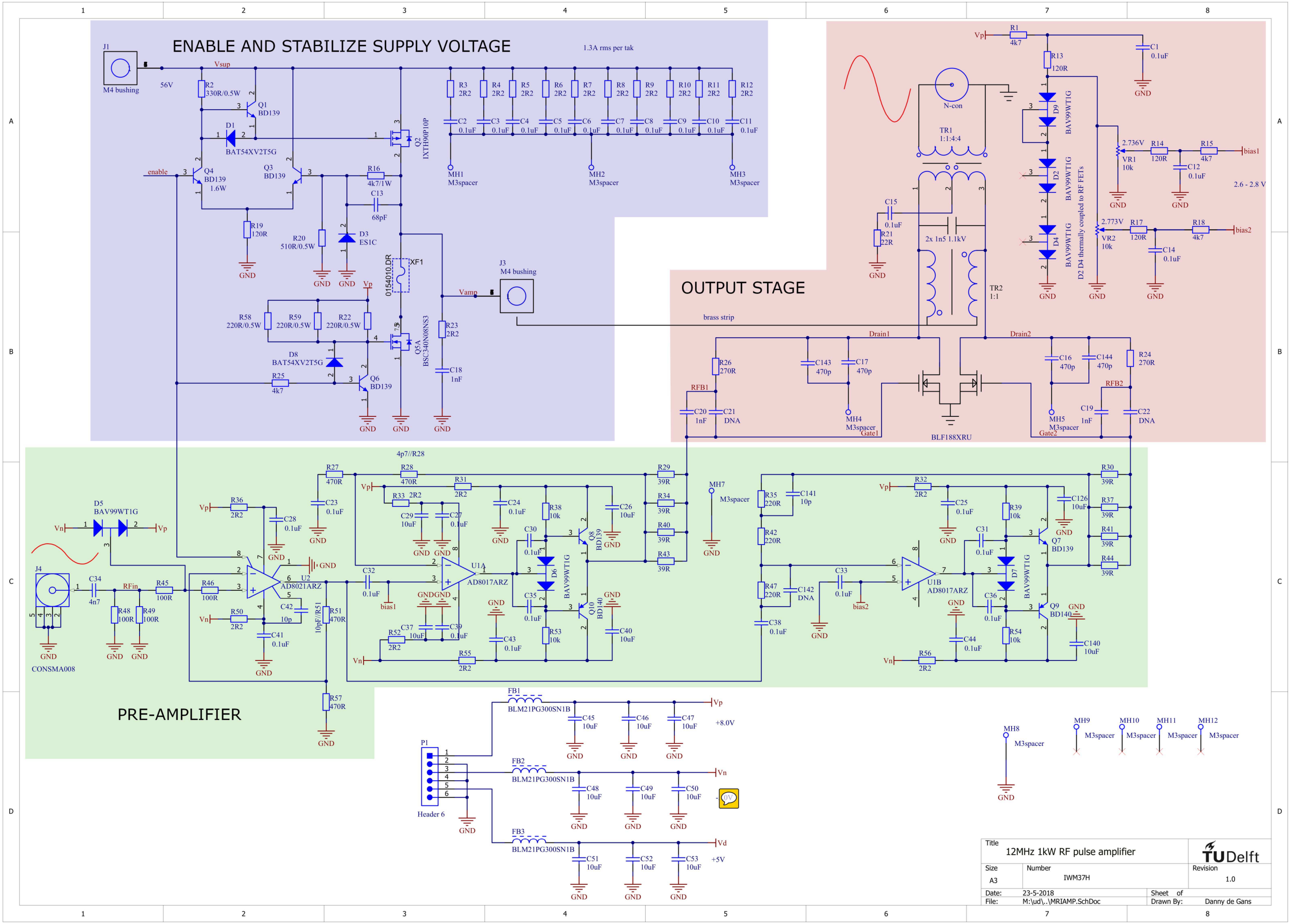

Supporting Information Figure S1. Schematic of a custom built 1 kW RF amplifier with the 3 main stages of the amplifier labeled.
