# Supplementary material for: In vivo 3D brain and extremity MRI at 50 mT using a permanent magnet Halbach array
Source: Magn Reson Med. 2020 Jul 5;85(1):495–505. doi: 10.1002/mrm.28396 (PMC7689769; doi:10.1002/mrm.28396)
Supplement: Supplementary file 2 — FIGURE S2 A 3D model of the x‐gradient coil wire pattern designed using the target field method described in Krishnan et al 31 [file MRM-85-495-s002.PDF]

Supporting Information Figure S2 - A 3D model of the X gradient coil wire pattern designed using the target field method described in [31].
